# Enviroscore: normalization, weighting, and categorization algorithm to evaluate the relative environmental impact of food and drink products

**DOI:** 10.1038/s41538-022-00165-z

**Published:** 2022-11-24

**Authors:** Saioa Ramos, Lucia Segovia, Angela Melado-Herreros, Maite Cidad, Jaime Zufía, Liesbet Vranken, Christophe Matthys

**Affiliations:** 1 AZTI, Food Research, Basque Research and Technology Alliance (BRTA). Parque Tecnológico de Bizkaia, Astondo Bidea, Edificio 609, 48160 Derio, Bizkaia Spain; 2 Clinical and Experimental Endocrinology, Department of Chronic Diseases, Metabolism and Aging, KU Leuven Leuven, Belgium; 3 Division of Bioeconomics, Department of Earth and Environmental Sciences, KU Leuven, Leuven, Belgium; 4 Department of Endocrinology, University Hospitals Leuven, Leuven, Belgium

**Keywords:** Sustainability, Environmental impact

## Abstract

A 5-scale label that relativizes the environmental impact of a given product referred to the impact of the European food basket is proposed. It was developed based on the Product Environmental Footprint methodology with the following stepwise approach. First, a set of normalization and weighting factors were defined to aggregate all the environmental impact categories into a single dimensionless index referred to as the European food basket, coined the European Food Environmental Footprint Single Index (EFSI). Next, the effectiveness of the EFSI index was evaluated by assessing the distribution of the EFSI results on 149 hypothetical food items and comparing it with the results obtained with EC Single Score. Finally, the thresholds to translate the EFSI index into the 5-scale Enviroscore (A, B, C, D, and E) were established and validated using the Delphi method. Results indicated that both, Enviroscore and EFSI, were able to account for impact variability between and within food products. Differences on the final score were observed due to the type of products (vegetables vs. animal products), the country of origin and the mean of transportation. Regarding country of origin, results indicated that differences in water stress impact category were better captured by the EFSI index (*r* = 0.624) than by the EC Single Score (*r* = 0.228). Finally, good agreement achieved with the Delphi method (weighted Kappa 0.642; *p* = 0.0025), ensures the acceptability of the Enviroscore. In conclusion, this study developed a method to communicate environmental impact assessment in a front-of-packaging label.

## Introduction

The planet is under unprecedented pressure. There is a growing body of evidence on how climate change, water scarcity, deforestation and pollution of ecosystems will compromise the capacity for nations to feed future generations^1–5^.

In this critical situation, food production and consumption have been reported as primary drivers of the human impact on the environment^6^. Food production accounts for 20 to 30% of the overall impact caused by human activities in the European Union^7^. In particular, food sector generates around 14 billion metric tons of carbon dioxide equivalents (CO_2_ eq) and it is responsible for the 26% of anthropogenic GHG emissions, 32% of global terrestrial acidification, and 78% of eutrophication^8^. Moreover, according to the Food and Agriculture Organization^9^, ~2.6 thousand km^3^ of water are consumed annually for agricultural purposes, 70% of the total water withdrawals.

Unlike other manufacturing sectors, food production is very heterogeneous in terms of efficiency, production practices, company size or seasonality^1,10^. Thus, the same final product could have different environmental performances depending on the origin or the production processes^8^. For instance, products with improvements in agricultural practices^11^, energy and water savings strategies^12^, food waste reduction^13,14^; or shorten distribution distances^15^ could significantly lower environmental degradation. Hence, a major change in the way food is currently produced and consumed is of tremendous importance to reduce environmental degradation and achieving Sustainable Development Goals (SDGs)^16–18^.

In this sense, Life Cycle Assessment (LCA)^19^ appears as a robust methodology for evaluating the overall environmental impact of a certain product or service and for identifying the potential environmental reduction due to the implementation of different environmental improvement strategies on manufacturing and supply-chain management^20^.

However, although assessing the life cycle of food products has been widely used for research or operational purposes, there are some limitations when communicating those results to final consumers:

- For instance, conventional approaches *only communicate one environmental impact categories*, being climate change usually communicated. Although studies concluded that carbon labeling could significantly reduce the carbon footprint of the food basket^21–23^, the benefit claimed may result in an undue transfer of impacts, ignoring the increase of other negative environmental impacts in the production chain.
- Other limitation with existing methods is that most of them focused on just one type of product. For example, in 2017^24^ demonstrated the effectiveness of communicating carbon footprint of milk to change consumer attitude. However, product-specific labeling does not allow comparison between products and thus, has a limited effect on the pursued radical change of dietary patterns. For instance, when providing category specific thresholds, it may introduce the perception that a ‘sustainable beef’ is less harmful for the environment than an ‘unsustainable banana’ although with the former having higher impact in all environmental impact categories.
- Additionally, existing environmental impact information systems of food products lack *a robust science-based method and have low scientific community support*^25^ and are usually reduced to a self-declaration (ecolabel Type III) with limited application to the consumers market^26^. For instance, the recently developed Eco-Score^27^. Even though it is based on average impact characterization results calculated according to LCA methodology, it considers a bonus-malus point system depending on the origin or private certification standards, among others. Although the method is publicly available, it has not been peer-reviewed yet.

The mentioned limitations make results of LCA incomparable and increases confusion for consumers. For instance, only about half of European consumers trust producers’ claims about environmental performance^28^.

In order to deal with these weaknesses, additional research on developing normalizing and weighting for a range of environmental impact categories is required in order to obtaining a single index which could suggest unequivocal results^29^.

According to the standard on LCA^19^, normalization is defined as “*calculating the magnitude of category indicator results relative to reference information*” and weighting as “*converting and possibly aggregating indicator results across impact categories using numerical factors based on value-choices*”. On the one hand, normalization can be used to compare the results with a reference situation that is external to or independent from the case studies, which may facilitate the interpretation and communication of the impact results^30^. On the other hand, weighting can facilitate decision-making in situations where trade-offs between impact category results do not allow choosing one preferable solution among the alternatives. The weights applied are supposed to represent an evaluation of the relative importance of impacts, according to specific value choices, reflecting preferences of, e.g., people, experts, or organizations, e.g. regarding time (present versus future impacts), geography (local versus global), urgency, political agendas or cost^31–33^.

This is the case of the Single Score^34^ developed by the European Commission (EC) in the framework of the Product Environmental Footprint (PEF) methodology^35^, where a set of normalization and weighting factors were put forward to calculate an aggregated final punctuation. In this case, the reference framework for the normalization values is based on the environmental impact of all goods and services of the European Union, considering both food and non-food. Within this broad universe of commodities, the relative environmental impact of a given food product is not well reflected, due to the noise caused by other non-food commodities, hindering between and within food products’ benchmarking possibilities.

Therefore, the goal of the current study is to develop new normalization and weighting factors to create a single index capable to reflect the relative environmental impact of food and drink products. Additionally, to facilitate the interpretation of the results by non-experts, cut-off values have been defined to create a 5-scale score. The ultimate ambition is that both methods, the aggregated index, and the 5-scale score, should be capable to demonstrate the relative environmental impact of their food choices in order to motivate them towards more sustainable consumption patterns and, simultaneously, to entice agri-food business to reduce the generation of environmental impacts throughout the supply chain.

In the next sections, we outline how the index, and the score were developed. First, we developed normalization factors (NF) using the environmental impacts characterization of the European Food Basket as a reference situation. Based on these new NF, we identified most recent and suitable weighting factors to create the single index for the Environmental Footprint of European Food and Drink products (EFSI) (section Development of European Environmental Footprint Single Index for Food and Drink Products). Afterwards, we verified that the EFSI addressed the capability to capture variability between different food products and within products (section Relative validation of the European Food Environmental Footprint Single Index). Finally, we established and verified threshold values to translate the EFSI index into an easy-to-understand 5-scale score (section Development and validation of the threshold values).

## Results

### European Food Environmental Footprint Single Index

**Fig. 1:**
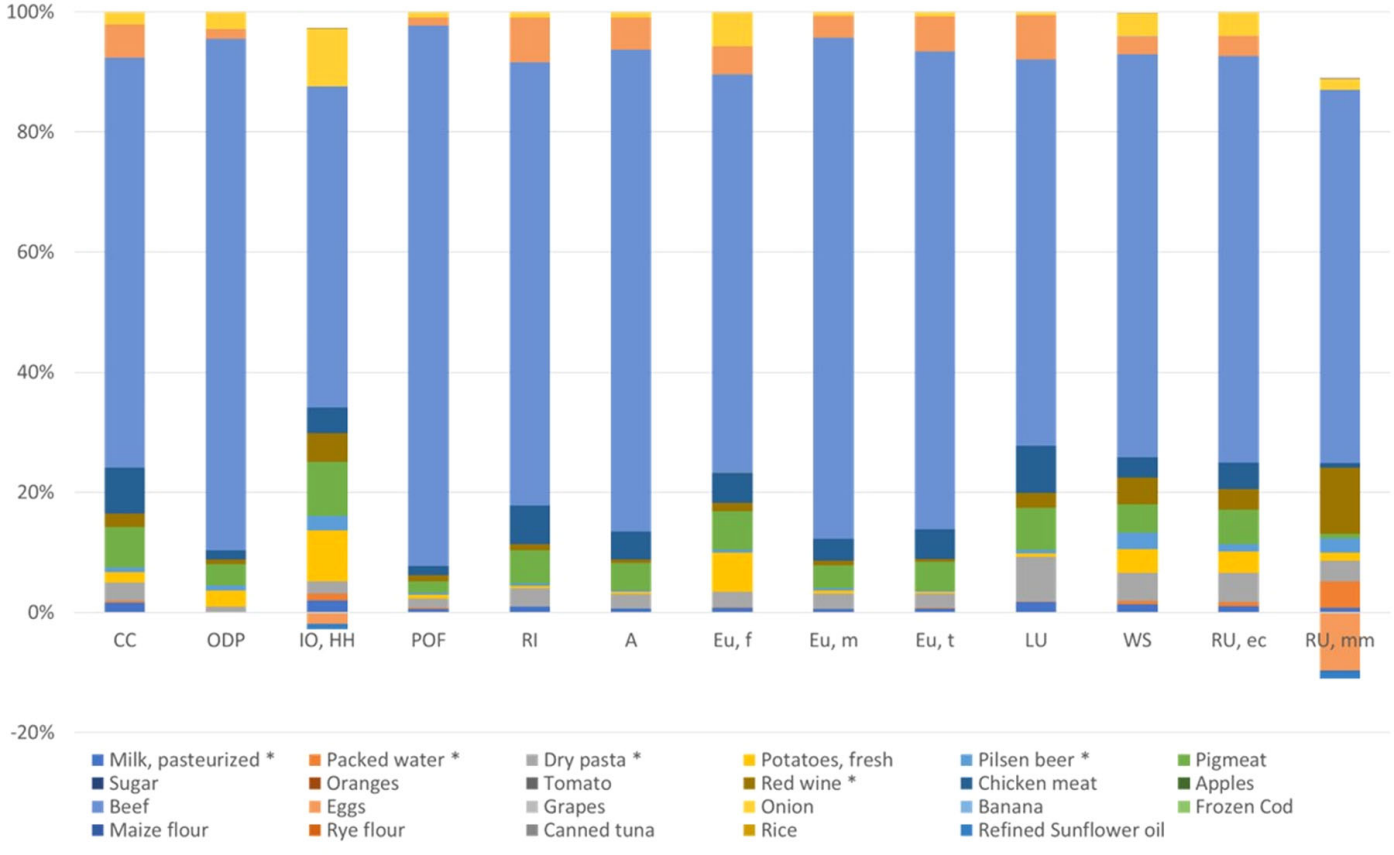
Environmental impact characterization including the 13 impact categories of the ILCD methodology of the representative food items of the European Food Basket. Where CC is climate change; ODP is ozone depletion potential; IR is Ionizing radiation; POF is photochemical ozone formation; RI is respiratory inorganics; ATF is acidification terrestrial and freshwater; EuF is eutrophication freshwater; EuM is eutrophication marine; EuT is eutrophication terrestrial; LU is Land Use; WS is water scarcity; and, RUe is resource use, energy carriers and RUm is resource use, mineral, and metals.

Figure 1 presents the environmental impact characterization results of the selected representative food items (*N*_1_ = 23). Those results will be used as reference universe for the NF of the EFSI method.

In the assessed European Food Basket, animal-based items comprise 28% of the total food consumption and overall contribute to 37% of the environmental impact. Within the animal-based food group, milk is consumed most (27%), while beef accounts for most of the environmental impacts (31% of the total impact). The 13 environmental impact characterization results of the European Food Basket including consumption (kg/year) are presented in Supplementary Material 1.

The impact characterization results of the European Food Basket were used as a baseline for the NF according to Equation 1(19). Both Normalization and weighting values of the EFSI are reported in Table 1.

**Table 1:** The European food environmental footprint single index normalization (EFSI-NF) and weighting factors^34^.

Impact category	Unit	EFSI-NF, per capita^1^	Weighting factor
CC	kg CO_2_ eq	2.42E + 03	22.19
ODP	kg CFC11 eq	1.29E-04	6.75
IR	kBq U-235 eq	1.31E + 02	5.37
POF	kg NMVOC eq	1.08E + 01	5.10
RI	disease inc.	2.44E-04	9.54
ATF	mol H + eq	3.93E + 01	6.64
EuF	kg P eq	3.81E-01	2.95
EuT	kg N eq	1.42E + 01	3.12
EuM	mol N eq	1.58E + 02	3.91
LU	Pt	2.43E + 05	8.42
WS	m^3^ depriv.	7.83E + 02	9.03
RUe	MJ	1.96E + 04	8.92
RUm	kg Sb eq	4.33E-03	8.08

^1^Global NF, per capita shall be used (Global population in 2013: 509,718,000 people).

Where CC is climate change; ODP is ozone depletion potential; IR is Ionizing radiation; POF is photochemical ozone formation; RI is respiratory inorganics; ATF is acidification terrestrial and freshwater; EuF is eutrophication freshwater; EuM is eutrophication marine; EuT is eutrophication terrestrial; LU is Land Use; WS is water scarcity; and, RUe is resource use, energy carriers and RUm is resource use, mineral, and metals.

### Relative validation of the European Food Environmental Footprint Single Index effectiveness

**Fig. 2:**
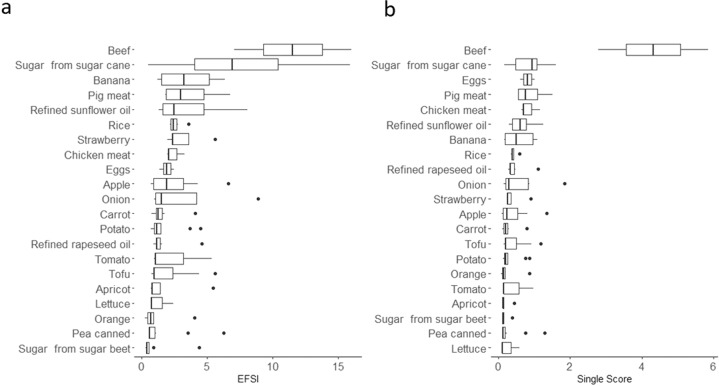
Distribution of the tnvironmental impact variability between and within the 21 representative food products. The figure shows the median and IQR of the EFSI (A) and EC Single Score (B) results of the *N*_2_ = 149 hypothetical food items of the 21 representative food products. The distribution of the impact characterization illustrates the variability between and within food products.

In general, the EFSI considerably varies between food products (Fig. 2A) as the EFSI of plant-based group was lower (median 1.30 (IQR 1.81)) than the EFSI of animal-based products (median 2.47 (IQR 4.21)). For instance, “sugar, from sugar beet” has the lowest EFSI median (0.379 (IQR 0.197)), while the highest is for “beef” (11.51 (IQR 4.48)). This trend could be found also in the EC Single Score for animal-based products (median 0.96 (IQR 0.81)) and plant-based products (median 0.24 (IQR 0.43)). However, the ranking of the specific products according to median environmental impact changed (Fig. 2A, B). For instance, in the example shown, the strawberry ranks the 7th according to EFSI, while for EC Single Score ranking, it is placed in the 11th position, from higher to lower impact. Moreover, the environmental impact variability within food products reported by the EC Single Score is lower than the variability reported by the EFSI (Fig. 2A, B).

**Fig. 3:**
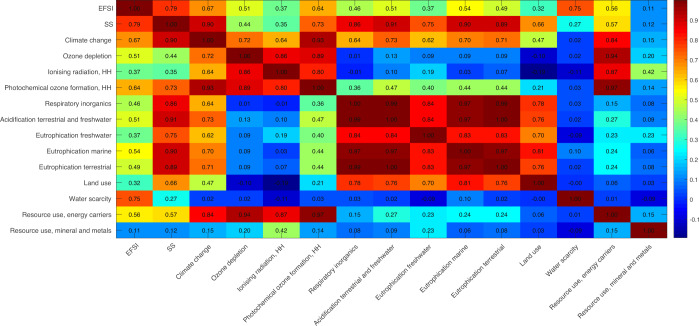
Heatmap representing the correlations between the 13 environmental impacts and the two single indexes, EFSI and EC single Score. The heatmap shows which impacts have higher correlation with each other and with each score.

Second, the correlation heatmap of the EFSI and the EC Single Score and their respective impacts categories was used to illustrate and compute the differences shown in the distribution of the impact characterization (Fig. 3). Regarding the correlation between environmental impacts, climate change has a good correlation (>0.60) with all environmental categories, except for water scarcity (0.02), resource use mineral and metals (0.15), and land use (0.47). Furthermore, water scarcity has very low to no correlation (≤0.11) with the rest of the environmental impacts, likewise for mineral and metal resource use (≤0.42). On the other side, correlations above 0.64 are reported among ozone depletion potential, ionizing radiation, photochemical ozone formation, and resource use (energy carriers). Moreover, similar correlations (≥ 0.69) are also found between respiratory inorganics, acidification, and freshwater, marine, and terrestrial eutrophication (Fig. 3).

Furthermore, regarding the correlation between environmental impacts with every single index, EFSI presents a medium correlation (≤0.67) with all the impacts, except for water scarcity, where the correlation is high (0.75). Additionally, EC Single Score shows high correlations to different environmental impacts, such as climate change (0.90), photochemical ozone formation (0.73), respiratory inorganics (0.86), acidification terrestrial and freshwater (0.91), eutrophication freshwater (0.75), eutrophication marine (0.90) and eutrophication terrestrial (0.89). However, a low correlation is found between EC Single Score and water scarcity (0.27). The overall correlation between EFSI and EC Single Score is high (0.79).

### Enviroscore: a new tool for food environmental footprint communication

After analyzing the details of the distribution of the EFSI results we established the threshold values (Table 2) in order to categorize the EFSI results into five scale score, the Enviroscore.

**Table 2:** Cutoff values and categorization of EFSI index considering the relative environmental impact of the food items.

Enviroscore	Environmental impact	EFSI
A	Very low	<4 × 10^−4^
B	Low	≥4 × 10^−4^
C	Medium	≥1.45 × 10^−3^
D	High	≥2 × 10^−3^
E	Very high	≥1 × 10^−2^

**Fig. 4:**
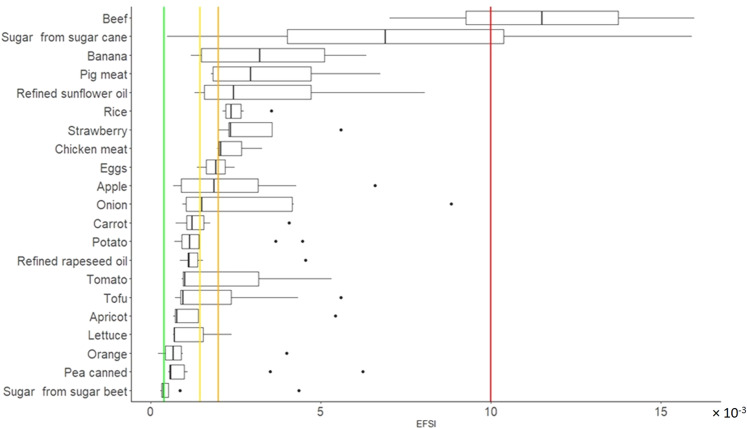
Distribution of EFSI result of the *N*_2_ = 149 hypothetical food items. Colored lines represent threshold values for the categorization. Being the green line the established threshold value (4.00 × 10^−4^) between very-low and low impact; Yellow line the threshold value (1.45 × 10^−3^) between low and medium impact; Orange line the threshold value (2.00 × 10^−3^) between medium and high impact; and Red line the threshold value (1.00 × 10^−2^) between high and very-high impact.

Food items with EFSI results below 4 × 10^−4^ have been considered as very low environmental impact. ‘A score’ food items encompass for example orange, rye flour, or soybean beverage. Products with EFSI values between 4 × 10^−4^ and 1.45 × 10^−3^ receive a ‘B score’, low environmental impact, which includes food items such as pasta, grapes, or potato. Food items with values between 1.45 × 10^−3^ and 2.00 × 10^−3^ are categorized as products with a medium environmental impact and receive a ‘C score’. For instance, fruit juices or refined sunflower oil can be found in this category. The ‘D score’ products include those with EFSI values between 2.00 × 10^−3^ to 1.00 × 10^−2^. In this category, we find high environmental impact food items such as avocado, chicken meat, or pig meat. Finally, products with EFSI values above 1.00 × 10^−2^, such as beef or canned tuna, have an ‘E score’, very high environmental impact (Fig. 4).

The validation of the accuracy of the Enviroscore classification with the categorization made by experts results in a good agreement (weighted Kappa 0.642; *p* = 0.0025). The contingency table (Table 3) shows 100% of accuracy for items categorized as A and E. However, at intermediate levels several deviations are reported between the classification based on the EFSI values which result in the Enviroscore, and the classification made by the experts. For instance, products with an Enviroscore B and D have a correspondence of 64% and 57% respectively with the expert-based categorizations. Finally, no agreement was reported for products categorized as C by the Enviroscore. Most deviations correspond only to movements of one level, backward or forward.

**Table 3:** Contingency table showing agreement between expert categorization (Delphi results) and Enviroscore.

Env. Score\Delphi	A	B	C	D	E
A	**100**%	0%	0%	0%	0%
B	21%	**64%**	14%	0%	0%
C	0%	67%	0%	33%	0%
D	0%	29%	14%	**57%**	0%
E	0%	0%	0%	0%	**100%**

Nevertheless, especially noteworthy is the case of strawberry product with an Enviroscore of B, which shows deviations of two levels. Experts participating in the Delphi round classified strawberry as B, while the Enviroscore classified it as D.

Finally, the validation of the performance of the Enviroscore was conducted. Based on the performance of the Enviroscore (Table 4), some deviations are found when comparing the accordance between the Enviroscore of the hypothetical alternative food items evaluated in step 2 and their respective representative food items (weighted Kappa 0.45, *p* < 0.05). Overall, the highest agreement is found in the items with Enviroscore B and D (62% and 78%, respectively) while items assigned to A and C present higher deviations from their representative food item, respectively 17% and 25%. Small deviations, switch off one level, are influenced by changes in the production methods (27%) and transportation (19%). This is the case of animal-based products, where beef could, for example, score E or D depending on production differences (beef from beef-cattle or from dairy cattle, accordingly). Large deviations, i.e., a switch of two or more levels, are mainly influenced by air (66%) and terrestrial transportation over large distances, especially in plant-based products. For instance, oranges, typically scoring A or B, are classified as D when imported from South Africa by plane, and apples, mostly categorized as B, score D when transported from China by lorry. Indeed, in a scenario without the items transported by plane, we obtain a weighted Kappa of 0.71, which indicates that transport mode highly influences the score that is assigned.

**Table 4:** Contingency table comparing results of the Enviroscore categorization of the representative products with the Enviroscore results of the hypothetical food item alternatives of each.

	Enviroscore of the hypothetical food item (*N*_2_)*				
Enviroscore of the representative product (N_3_)*	A	B	C	D	E
A	17%	67%	0%	17%	0%
B	4%	62%	7%	27%	0%
C	0%	17%	25%	58%	0%
D	0%	0%	22%	78%	0%
E	0%	0%	0%	50%	50%

*Only products that were present in both data set (representative and food item scenarios) were included. The food item scenarios (initially *N*_2_ = 149) were 130 after the exclusion of sugar from sugar cane (*n* = 13), apricot (*n* = 5) and a cultivar of lettuce (*n* = 1). The representative products (initially *N*_3_ = 22) were 19 food items after the exclusion of three food products due to the lack of data to build the hypothetical scenarios.

All results of the impact characterization, normalization, and endpoints of EFSI and EC Single Score, and Enviroscore of all the food items studied are shown in Supplementary Material 1 (SP1).

## Discussion

In this study, we present normalization and weighting factors for the new Single Index adapted to the European food and drink sector. The single index, so-called EFSI, provides a science-based approach that combines 13 environmental impact categories into one value. Furthermore, we propose threshold values to transform the absolute EFSI values into an easy-to-understand 5-scale score, the Enviroscore.

The Enviroscore provides a summarized and relative information of the environmental impact of food items. The Enviroscore is based exclusively on PEF compliant environmental impact categories, accounts for environmental impact variability between and within food products.

Variability captured by the EFSI and Enviroscore shows to be selective and sensitive. As expected, plant-based products have been categorized as low impact products^8,36^. The developed score is also able to discriminate according to differences in distribution mode (local vs international air)^15,37–39^. Moreover, differences in production environment (greenhouse vs field production) are found as well^38,40^. reported significant differences between strawberry produced in heated and unheated greenhouses, which is also captured by the Enviroscore. Differences according to the country of origin are identified, particularly for countries that have been reported as water stressed, such as Spain or Pakistan^41,42^. The EFSI and Enviroscore could differentiate between beef from beef-cattle and beef from dairy-cattle, which have been previously identified as substantial differences in impact^8,43^. Additionally, the Enviroscore does not change when comparing organic and conventional produced foods, as found as well by^44^.

Regarding the validation of the accuracy and performance of the Enviroscore, the deviations observed were expected due to the absence of a golden standard for the interpretation of food environmental impacts and because of the inherent variability of environmental impacts related to food products. Nevertheless, the Enviroscore shows a good level of agreement.

A potential source of bias is the environmental impact characterization results of the European Food Basket, the baseline used for the NF. When the environmental impact results of our European Food Basket are compared with the recently published EU (Food) Basket of Products^45^, hardly any difference could be found. Climate change, acidification, land use, respiratory inorganics and resource use, energy carriers potential impact categories vary less than 20% when comparing both studies, while we observe higher differences in the magnitude order in terrestrial eutrophication, ionizing radiation, and photochemical ozone formation. Since minor differences are reported on the assumptions made for the inventory analysis, source of differences between the two baskets falls on the selection of relevant products. For instance^45^, included cheese, butter, and salmon in the food basket. These are products with high impact on terrestrial eutrophication, ionizing radiation, and photochemical ozone formation. Those products are not included in our basket since we selected just one representative food item for each food category as defined by^46^. Hence, in our study, we considered cod fish, and not salmon, as representative of the demersal fish. Similarly, for dairy products we selected milk as the representative since it is the most consumed dairy product, without considering cheese. Moreover, despite the fact that FAO classifies butter as “Fat and Oils”, butter was neither selected because its low contribution to the overall EU food basket. According to^45^, butter represents only 0.6% of the basket. However, although substantial, those differences would not alter the final EFSI results since those impacts, terrestrial eutrophication, ionizing radiation, and photochemical ozone formation, have 5% or less of the weighting factor.

Other potential limitations of the EFSI index could be that the final values would significantly deviate from the EC Single Score. Nevertheless, in our study we prove that EFSI does not differ significantly from the EC Single Score, and main differences are attributed to the higher correlation of the EFSI with water scarcity impact category. Indeed, all the environmental impact categories are moderate to highly correlated with both EFSI and the EC Single Score, except from water scarcity. The EC Single Score considers the consequences of water withdrawal but only to a limited extent. Moreover, water scarcity is a stand-alone impact category that is poorly correlated with all the other impact categories. Water consumption is of great importance in the food industry, and more specifically in agriculture and livestock. For instance, farming accounts for almost 70% of all water withdrawals, and up to 95% in some developing countries^9^, while in Europe agriculture accounts for almost 40% of the total water withdrawal^47^. As food production is a major contributor to water scarcity^9^, it is important to ensure that the proposed index considers distinctively potential impact derived from water consumption, specifically in those countries or river basins with high water stress levels. Hence, it is of a high importance that the Enviroscore evidence, particularly products utilizing high amounts of water or produced in countries with high water stress.

The selected functional unit for the food product EFSI and Enviroscore evaluation appeared as potential constraint. To compare the environmental impact across products, LCA methodology requires a functional unit that captures the obligatory properties of a product. However, functional units of food products are difficult to define due to variation in perceived obligatory properties^48^. Most studies proposed a mass-based functional unit, while some authors proposed protein content and quality, energy content, or nutrient density as the basis of the comparison^8,49–51^. In the EFSI methodology we select a functional unit based on the mass of the final product, following the rules established by the PEF guidelines, where mass-based functional units are defined. However, in order to make it comparable, we select 1 kg of product as functional unit for all the product types, without considering different portions that the PEF guidelines considers, such as 1 hl of beer and 100 ml of packed water. This selection is also aligned with the current mandatory food labeling since it is easy to compare among products. Different types or amounts of functional unit per food products would have introduced more uncertainties to end-consumers and decreased the comparability between food products^52^.

According to the single market for green products initiative, all food products commercialized in Europe should evaluate their environmental impact following the recommended PEF methodology^35^. The calculation of the PEF requires a lot of personal and economic effort. Currently, the main barrier for the transition of the method is that companies could not use the obtained results to add value to their products since there is no clear strategy to communicate the environmental impact characterization results obtained with this methodology. Thus, current method represents a step forward also to encourage food companies to evaluate their environmental footprint. Providing a unique EFSI and Enviroscore per product and food company would allow companies to benchmark their products according to their real environmental impact. Additionally, with the Enviroscore, we aim to raise awareness on the actual environmental impact of food products and to contribute to the mitigation of food environmental burden while food preferences and cultures are maintained. As it is shown in the study of^53^, increasing consumer knowledge about the environmental impact of food choices could lead towards more responsible consumption patterns. Consequently, higher demand of environmental-friendly food products could incentivize food industry to improve their practices. This assumption is in line with the Energy label of consumer electrics in EU^54^ and the nutritional labeling initiatives^55^. Both initiatives introduce, respectively, an optimization of efficient energy use and a food product reformulation. To conclude, from a policy point of view, the Enviroscore can be used to communicate in an easy-to-understand way the environmental impact of food products and as such food system actors will be encouraged to implement the best environmental practices within their companies.

According to the authors’ knowledge, the proposed methodology could be used to communicate the relative environmental footprint of foods and drink products in a transparent and science-based manner. The methodology is unique as it is based on European PEF methodology and reflects between and within food product variability in environmental impacts.

Nevertheless, the current results are based on a limited number of data points and food products. In order to further evaluate the robustness and sensitivity of the proposed EFSI and Enviroscore additional case studies should be included in the analysis.

## Methods

**Fig. 5:**
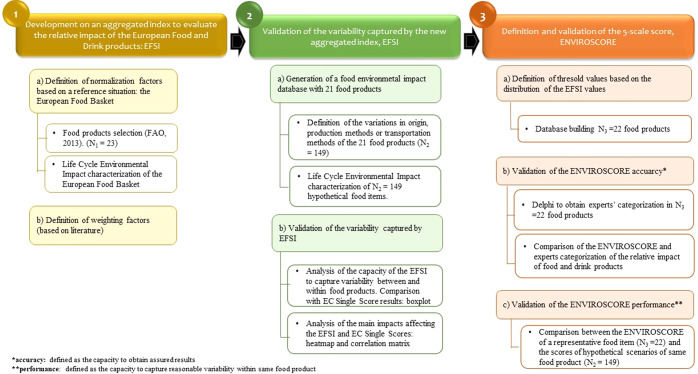
Scheme representing the main steps of the methodology followed to develop and validate the Enviroscore. First, the development on an aggregated index to evaluate the relative impact of the European Food and Drink products (EFSI) was created. As a second step, the variability capture by EFSI was validated. Finally, the Enviroscore was defined as a 5-scale score.

A stepwise approach was used to develop a single index and a score (Fig. 5).

### Development of European Environmental Footprint Single Index for Food and Drink Products

According to^19^, the aggregation of the environmental impacts to calculate a single index of a product requires a set of normalization and weighting factors, one for each of the environmental impact categories.

In this study, we developed NF defining as a reference situation the environmental impact linked to the average European food basket.

The European food basket is defined as a representative group of food and drink products purchased yearly by European citizens it considers food products that together constitute 90% of the total food supply in Europe (EU-28). The food products representing the basket were selected from the FAO Food Balance Sheets^46^. Additionally, packed water was added to the final product list, due to its high consumption in EU^7^. In total 23 food products were identified (*N*_1_ = 23). For each product, a representative food item was defined reflecting the most common production practices, origin, distribution ways, consumption patterns, and end-of-life scenario.

Upon defining the reference situation, we calculated the environmental impact of the European food basket following the Product Environmental Footprint methodology as recommended by the EC^35^. For the inventory data required to calculate the impact of the European food basket we considered the following data sources, assumption, and limitations:

i. Origin and primary production: Considering Eurostat import-export rates, background datasets reflecting the country of origin of the primary production stage of the selected products were selected from Ecoinvent 3.5^56^ and Agri-footprint datasets^57^. For the products with high importing rates and variety of origins, datasets representing global statistics were selected.
ii. Food processing data (water, energy, and refrigerant consumption) for fresh vegetables was selected from^58^ and TeslaProject^59^. Inventory data for processing cereals and animal-origin products were obtained from Ecoinvent 3.5^56^ and Agri-footprint datasets^57^ European average datasets.
iii. Data regarding packaging solutions for each type of product were defined based on^7^.
iv. International distribution was included when more than 20% of the supply of the product was imported from outside the EU^46^. Data was retrieved from^60^, including country of origin and mean of transportation. Distances between the country of origin and the EU were based on a standard entry point by boat (Rotterdam, The Netherlands), by plane (Frankfurt, Germany), or by train and lorry (Rungis, France).
v. National distribution was assumed to be 500 km by lorry for all products^7^.
vi. Data on storage energy, water, and refrigerant consumption by retailers was defined based on^61^.
vii. Inventory data related to consumer transport (both home delivery and own transport), cold storage or freezing, preparation/cooking, and bio-waste management were selected from^61^.
viii. Finally, inventory data for the end-of-life of packaging was obtained from^62^.

Background datasets for water and energy supply, transport carriers, and waste disposal were retrieved from the Ecoinvent 3.5 database^56^. For pasteurized milk^63^, packed water^64^, dry pasta^65^, Pilsen beer^66^, and red wine^67^ environmental impact results published by the EC were used.

Life Cycle Inventory and selected datasets for the representative items used to build the European Food Basket are available in Supplementary material 2 (SP2).

We used Simapro 9.0^®^ software^68^ to calculate the 16 environmental impact categories of the European Food Basket recommended by the EC^35^ following the impact characterization methodology of the International Reference Life Cycle Data System (ILCD)^69^. The ILCD considers climate change potential, ozone layer depletion, eutrophication, acidification, or water stress index among other environmental impacts.

The environmental impact characterization results of the European food basket (23 representative food items) were used as the reference situation to define the new NF for each environmental impact category in line with^1,70^

1 $$NF_{(i)}{{{\mathrm{ = }}}}\frac{{\mathop {\sum }\nolimits^ FC\left( i \right) \times e(f_i)}}{{Population}}$$

Where *NF* is the normalization factor per capita; *f*_*i*_ is a representative food item included in the European food basket (with *i* = 1, …23); *FC(i)* is the yearly European consumption (in kg) of food item i; *Population* is the number of EU-28 inhabitants; and *e* equals the environmental impact per kg of food item and refers to one of the following environmental impact categories recommended by the ILCD methodology: climate change (kg CO_2_ eq.), ozone depletion (kg CFC-11 eq.), ionizing radiation (kBq U-235 eq.), photochemical ozone formation (kg NMVOC eq.), respiratory inorganics (disease inc.), acidification terrestrial and freshwater (mol H + eq.), eutrophication freshwater (kg P eq.), eutrophication marine (kg N eq.), eutrophication terrestrial (mol N eq.), land use (Pt), water scarcity (m^3^ eq.), resource use, energy carriers (MJ), resource use, mineral and metals (Kg Sb eq.).

Once the NF were defined, we identified the most suitable weighting factors for the aggregation of the 13 environmental impacts. As already stated, the applied weights were supposed to represent an evaluation of the relative importance of impacts reflecting different aspects, such as, opinions, geography, or political agenda. For this approach, we selected the weighting factors developed by^34^ recommended by the EC. This weighting factors dismissed the impact categories related to toxicity (human toxicity cancer and non-cancer effects, and ecotoxicity) due to the lack of robustness of the methodologies to calculate those impacts.

The aggregation of the environmental impact categories resulted in the EFSI. This single index provides information on the environmental footprint of food items, relative to the average per capita European food consumption impact.

### Relative validation of the European Food Environmental Footprint Single Index

After the calculation of the normalization and weighting values, the main objective was to evaluate whether the EFSI index reflected the variability between the impact of different food products and the variability within products (same product but items with changes in the production methods and origin).

For the validation, we analyzed the differences on the distribution of the EFSI and EC Single Score results of different food product^34^. Additionally, we assessed differences on the sensitivity of the two indexes to individual environmental impacts.

For that purpose, we created a second dataset containing 21 food products and for each one a range of hypothetical food items (*N*_2_ = 149). These hypothetical items were defined considering the current diversity in the origin of the product, transportation ways, and differences in production methods of the food items consumed in the European market^60^. For example, for “Potato” product, a variety of 11 hypothetical “potatoes” were considered including conventional, organic, or integrated potato production methods and distribution from China, Ukraine, Russia, or USA among others. Supplementary material 3 (SP3) provides information on inventory data of the 149 food items.

The relative validation was carried out as follows:

- First, in order to understand the capability of each score to capture the variability between and within products, we compared the distribution of the EFSI results and the EC Single Score results of the *N*_2_ = 149 food items. To this purpose, a boxplot was used to visualize the range, interquartile range (IQR), and median of the EFSI and the EC Single Score results.
- Second, to understand the reason behind the potential differences observed between the EFSI and the EC Single Score distribution we analyzed which impacts have more influence on each of them (EFSI and EC Single Score) and which impacts are correlated to another. For such purpose, we built a correlation heatmap using the impacts data and the EFSI and EC Single Score results of the *N*_2_ = 149 food items to visualize a 2D correlations matrix between the environmental impacts results and EFSI and EC Single Score. This type of visualization graph uses colored cells to represent data where the color of each cell is proportional to the correlation that matches the dimensional value.

We carried out all the statistical analysis with RStudio (version 1.1.463) and Matlab 2017b (The Mathworks, Inc). For the correlation heatmap the code from^71^ implemented on Matlab 2017b (The Mathworks, Inc.) was used.

### Development and validation of the threshold values

Step 3 is the definition and validation of the EFSI threshold values to establish a 5-scale score, coined the Enviroscore.

We defined the thresholds based on the distribution pattern of the EFSI. In line with the methodology used in the development of the Nutri-Score^72–75^. In order to reflect the environmental impact distribution of the food product of the current and future market, an additional dataset was collated containing 22 representative food items (*N*_3_ = 22). This dataset encompasses the 12 representative food items of the European Food Basket and an additional set of 10 food items including food products with a steep increase the market demand to account for potential changes in food acquisition trends. The EFSI of the additional food items was calculated as described in Step 1 (see SP1).

Afterwards, the accuracy of threshold values was validated., namely the capability of the Enviroscore to obtain assured results. In the absence of a golden standard, we compare the obtained Enviroscore results on the relative environmental impact of the food products with experts’ categorization. To obtain experts' agreement on the relative environmental impact of food items we used the Delphi method. All the information regarding the protocol, questionnaire, and results are available in Method section. In summary, the Delphi method is a technique used to gain insight into a particular topic. It uses an iterative feedback technique with a group of experts, and it has been widely used to gather expert’s opinions and consensus in numerous fields, such as identification of food safety priorities^76^, definition of policies to improve population nutrition^77^ or the identification of key factors affecting e-commerce^78^. For the purpose of the study, we defined ‘expert’ as those who have at least three years of experience in calculating environmental impact of food items, or at least one year of experience working with life-cycle assessment method to calculate environmental footprint of food items and have working experience in European food products. The Delphi method was carried out with seven experts from February till April 2019. It consisted of three rounds of expert’s feedback through a series of semi-anonymized online surveys. Experts categorized 22 food items as very low-, low-, medium-, high-, and very high-impact. Food items were withheld only when 80% of the experts agreed in the categorization. Their categorization was then compared with the Enviroscore results of the representative products (*N*_3_ = 22). We evaluated the agreement between the expert categorization of the food products and the categorization of the Enviroscore by calculating the weighted Kappa, a coefficient that signals the overall agreement^79^. Indeed, we evaluated the accuracy of the categorization between the Enviroscore results and the experts’ opinion by a contingency table that portrays the coincidences between the categorization by Delphi experts and by the Enviroscore when assessing the same product.

Finally, we tested the performance of the categorization, namely the capacity of the Enviroscore to capture reasonable variability within the same food product. To do so, the Enviroscore results of each representative food item (*N*_3_ = 22) was compared with the results of the same hypothetical food item (*N*_2_ = 149). In example, the score of a representative orange is compared with the scores of hypothetical oranges (e.g., orange produced in China and transported by plane). For that comparison, a second contingency table was built to represent the coincidences and variations between the Enviroscore results of the 22 representative food items and the scores of each hypothetical item.

### Delphi method protocol

Delphi protocol followed in this study required the definition of steps and criteria that will be followed. The following steps and criteria that ensure the reliability and validity of the protocol were approved by the Katholic University of Leuven ensuring also that the protocol complied with all relevant ethical regulations:

i. **Identification and selection of the experts**. The first step for identification and selection of the experts is defining the criteria of what an expert is and determining the person’s field of expertize. For our study: (i) at least three years of experience in calculating environmental impact of food items, (ii) at least one year of experience working with life-cycle assessment method to calculate environmental footprint of food items, (iii) work experience in European food products.
  Once expert definition and criteria were set, we proceeded to identify possible experts. The identification was done by searching on LCA conferences attendee list and by brainstorming between researchers involved in this procedure. Afterward, all the information about possible experts was collated in a database, that contained the following information: name, country, organization, expertize, email, person of contact and potential conflict of interest. The targeted minimum number of experts is *n* = 10.
ii. **Identification and selection of the food products**. The food items selected for this study are representatives of the main food groups consumed by European citizens^80^. In addition, we included two additional food groups: “Fish and Seafood” and “Legumes and Legumes-based products”. Both groups are growing markets in EU^81^, and represent an important source of environmental degradation and an alternative to animal-based protein, respectively. Food products and food groups were categorized according to FoodEX2 food category system^82^.
iii. **Selection of the number of rounds**. The number of rounds in the Delphi method should be enough to reach consensus without tiring the experts. Hence, defining consensus is important to mark the end of the Delphi rounds. The end of the Delphi was set when reaching experts’ consensus (agreement between experts’ opinion above 80%) for all food products, with a maximum of three rounds.
iv. **Statistical analysis**. We analyzed the data performing simple descriptive analysis, such as median, distribution (IQR) using R version 3.5.2. Furthermore, the percentage of agreement with each food product was calculated to identify where the sources of disagreement were.
v. **Reporting results back to experts**. Reporting the results back to the experts is a necessary step to reach consensus. The data reported back contained a ranked list of food items according to the environmental impact category (Low to High Impact) and the level of agreement (0 to 100%).
vi. **Recruiting the panel**. Preselected experts were contacted via email to introduce the study and to request their participation, including information about the Delphi method, estimated time needed for successful completion and what and how the data provided would be managed. Moreover, we remarked the commitment throughout the several rounds. (83) Two reminders were sent after a week and 2 weeks without response to increase the response rate. An informed written confirmation was obtained from all the participants who accept being part of the study. In order to ensure the anonymity of the expert, the names were coded, and questionnaires were filled with their unique code instead of experts’ names.
vii. **The Delphi Round**.
  ***Round 1****. Preparing the questionnaire:* The online questionnaire was generated using Survey Monkey. The content of the questionnaire was decided by agreement between the researchers from AZTI and KUL involved in this procedure. The first round of questions, Questionnaire 1 consisted of two sections: a) Section one contained introductory information about the questionnaire and some questions regarding personal information. b) Section two was the body of the questionnaire. In this section first, a general statement regarding considerations to categorize food products was shown, followed by a selection of 38 food products that needed to be categorized by its environmental footprint. The experts had to rank all the food products from low to high impact in a 5-point Likert-scale (corresponding to A to E categorization) according to the relative environmental footprint, according to their experience. No information was provided about the environmental footprint, nor about the production system, only information to differentiate one food product from another. Two open boxes were provided both at the end of each food group and at the end of the questionnaire to collect feedback of the rationale behind each answer and about the overall questionnaire performance respectively.
  A small group of experts (*n* = 4) from Azti (https://www.azti.es/en/) and Corluyt (https://www.colruyt.be/) piloted the questionnaire (Q1) beforehand. This pilot ensured that information was clearly stated and there was a minimum error in the design of the questionnaire. After the pilot, minor adjustment was done, and the questionnaire was sent to the selected experts.
  ***Round 1****. Transmitting the questionnaire:* Upon participation agreement, questionnaire 1 was sent through email to each expert. The same follow up system as during recruitment was followed. Hence, experts were contacted after a week and two weeks of no response.
  ***Round 1****. Analyzing the results:* Once the results were received from experts we performed statistical test as described in the section “Statistical analysis”. Afterward, a report was prepared and sent together with the next round of questionnaire to the experts.
  ***Round 2****. Preparing the questionnaire:* During the round 2 of questionnaires, questionnaire 2 (Q2) had similar structure as Q1 with modifications only in the Section two. Modifications were done according to the results and comments received in the previous round.
  ***Round 2****. Transmitting the questionnaire:* In Round 2, questionnaire 2 (Q2) was sent together with the report of the results of the previous round (R1) and some additional information regarding each food product. The same follow up system as in previous steps was followed. Hence, experts were contacted after a week and two weeks of no response.
  ***Round 2****. Analyzing the results:* Results were analyzed statistically as described in section “Statistical analysis”. We compared the results obtained from round 1 with the results of round 2. After analyzing the results, we decided we had to follow with the next round. A report was sent together with the next round of questionnaire.
  ***Round 3****. Preparing the questionnaire:* During the final round (R3) of questionnaires, questionnaire 3 (Q3) had similar structure as Q2 with modifications only in Section two. Modifications were done according to the results and comments received in the previous round.
  ***Round 3****. Transmitting the questionnaire:* In Round 3, questionnaire 3 (Q3) was sent together with the report of the results of the previous round (R1). The same follow-up system as in previous steps was followed. Hence, experts were contacted after a week and two weeks of no response.
  ***Round 3****. Analyzing the results:* Results were analyzed statistically as described in section “Statistical analysis”. We compared the results obtained from round 2 with the results of round 3.
viii. **Reaching consensus**. The consensus aims to provide the food categorization (A to E score) of all food products presented to experts. A final report was sent to the experts, together with a thank note for their participation and notifying the ending of the Delphi method.

## Supplementary information

Supplementary Material

## Data Availability

The authors declare that all data and results generated or analyzed during this study are included in this published article and its supplementary information files.
